# First person – Samantha Thompson

**DOI:** 10.1242/dmm.052512

**Published:** 2025-06-23

## Abstract

First Person is a series of interviews with the first authors of a selection of papers published in Disease Models & Mechanisms, helping researchers promote themselves alongside their papers. Samantha Thompson is first author on ‘ P23H rhodopsin accumulation causes transient disruptions to synaptic protein levels in rod photoreceptors in a model of retinitis pigmentosa’, published in DMM. Samantha is a PhD candidate in the lab of Dr Michael Robichaux at West Virginia University, Morgantown, WV, USA, investigating protein trafficking with high-resolution retinal imaging.


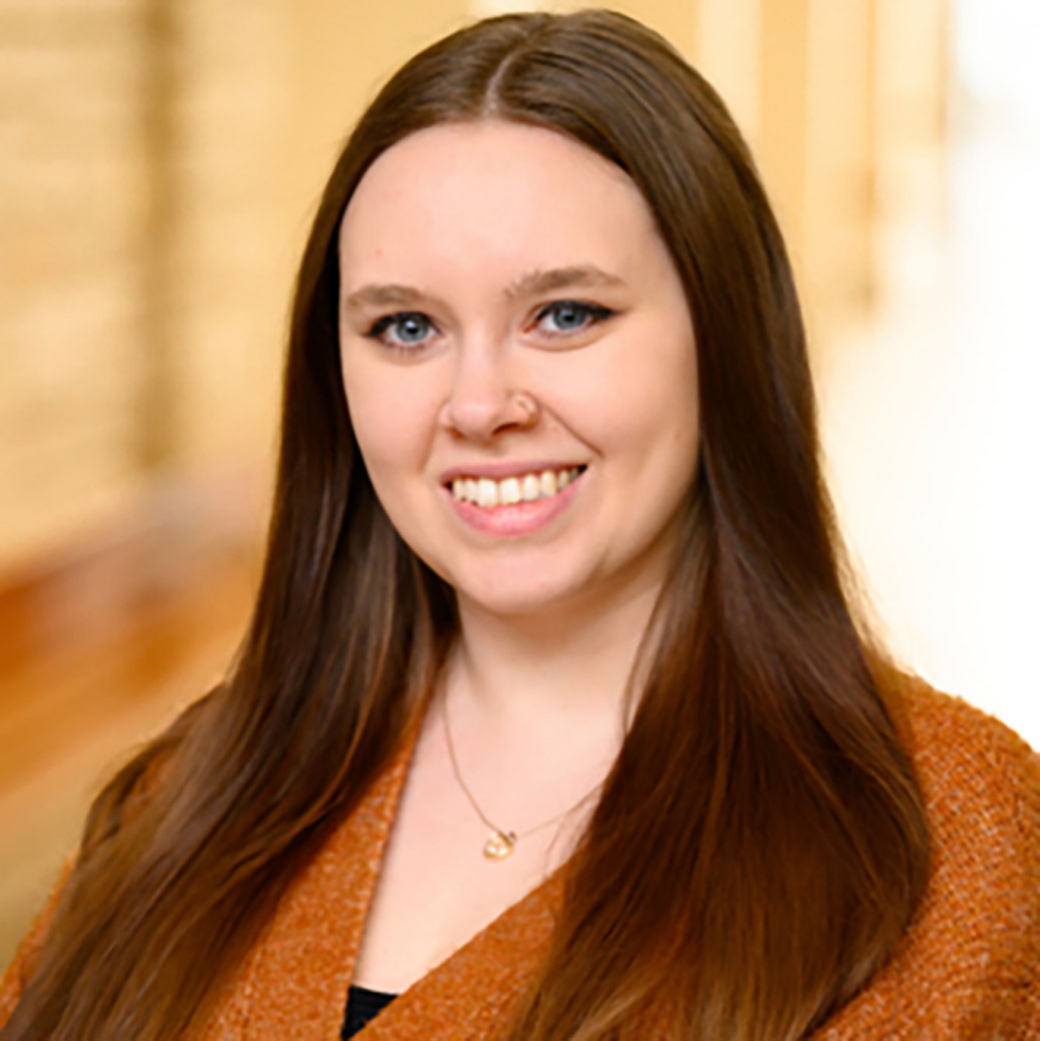


**Samantha Thompson**

**Who or what inspired you to become a scientist?**

During my senior year of high school, there was an Ebola outbreak. My science teacher that year had us watch documentaries about Ebola, which inspired me to look more into becoming a scientist. I ended up switching my college major from theatre and music to microbiology with the intention to apply to med school. Then, during my undergrad, I got the chance to do research, which solidified my decision to pursue being a scientist instead.

**What is the main question or challenge in disease biology you are addressing in this paper? How did you go about investigating your question or challenge?**

The main question is how rod photoreceptor synapses are impacted during diseases like retinitis pigmentosa. My PI's postdoc work led to a mouse model where mislocalized rhodopsin could be seen at the synapse, so this study looked further into this mislocalization and what the impact was on the synapses.

**How would you explain the main findings of your paper to non-scientific family and friends?**

I always just tell my family and friends that I look at mouse eyes to see what happens when a protein goes where it shouldn't be and that I use fancy microscopes to take pictures. I often refer to myself as a synapse photographer.

By gaining a better understanding of how rod synapses are impacted in disease, we could potentially be able to identify better therapeutic windows to provide treatment before synaptic connections are lost.

**What are the potential implications of these results for disease biology and the possible impact on patients?**

By gaining a better understanding of how rod synapses are impacted in disease, we could potentially be able to identify better therapeutic windows to provide treatment before synaptic connections are lost.

**Figure:**
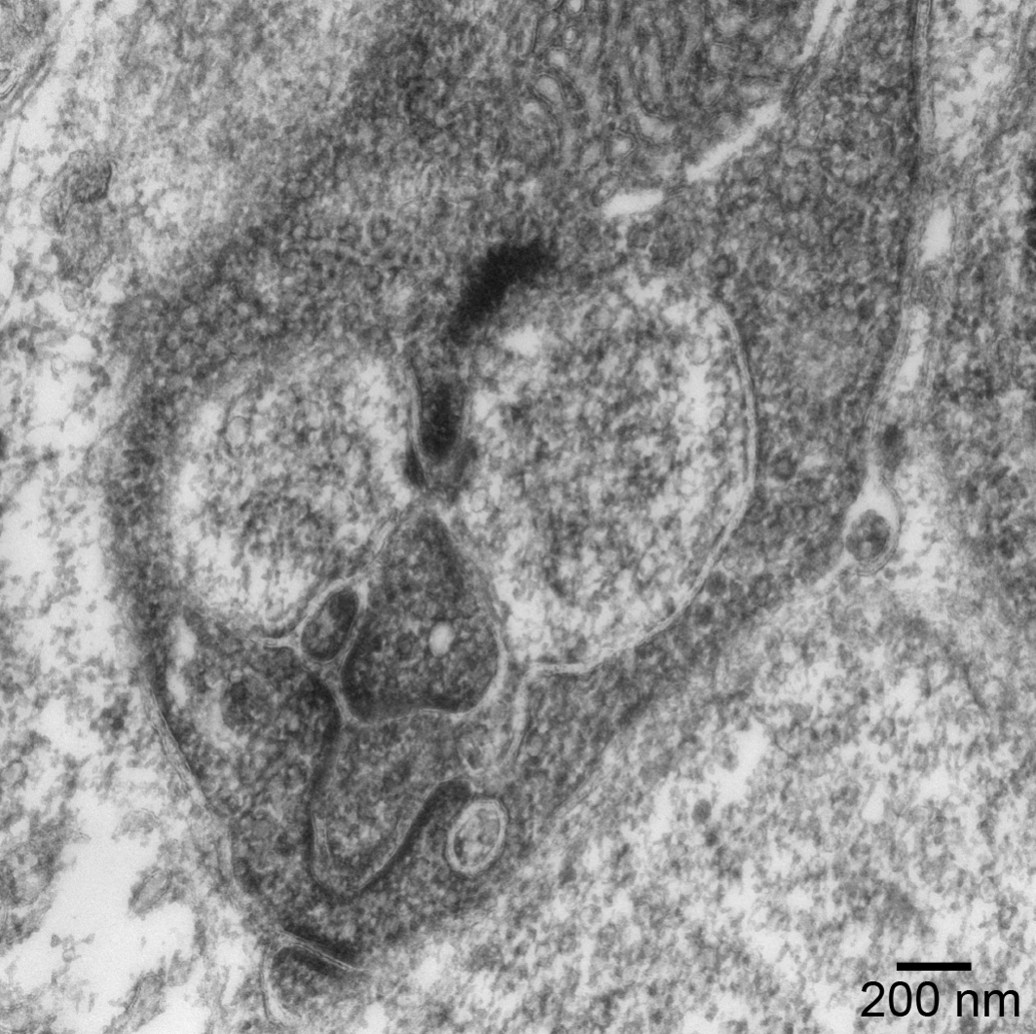
**The worm-face synapse.** This wild-type mouse rod spherule looks like a worm but also has a face. Image was acquired by transmission electron microscopy.

**Why did you choose DMM for your paper?**

DMM is the journal that published the paper that validated my primary mouse model. Also, DMM publishes some excellent work that is relevant for disease and health and seemed like an overall good fit for the paper.

Communicating with the general public is becoming increasingly important, and social media can be a valuable tool.

**Given your current role, what challenges do you face and what changes could improve the professional lives of other scientists in this role?**

I feel like social media training and science communication should be taught more in graduate school. Communicating with the general public is becoming increasingly important, and social media can be a valuable tool. Public opinion regarding research and what should be funded has been an incredibly hot topic lately, and the futures of grad students that are hoping to take the next steps soon are becoming more unclear and hang in the balance. By better communicating science, we can work to combat misinformation and make things better for future scientists.

**What's next for you?**

I'm going to be doing a postdoc with Dr Will Spencer at SUNY Upstate Medical University. I'm very excited to move to Syracuse for the next step of my career and to be mentored by the wonderful faculty there.

**Tell us something interesting about yourself that wouldn't be on your CV**

I have a background in musical theatre. I took private voice and piano lessons as a child, was in the school choir and participated in musicals and plays. I'm currently in the community choir at West Virginia University so that I can still sing.
